# Gastrointestinal Parasite Infections and Environmental Sustainability of the Ovine Sector: *Eimeria* spp. Infections and Nitrogen and Phosphorus Excretions in Dairy Sheep in Italy

**DOI:** 10.3390/pathogens12121459

**Published:** 2023-12-16

**Authors:** Irene Sodi, Mina Martini, Federica Salari, Stefania Perrucci

**Affiliations:** 1 Department of Veterinary Sciences, University of Pisa, Viale delle Piagge 2, 56124 Pisa, Italy; irene.sodi@phd.unipi.it (I.S.); mina.martini@unipi.it (M.M.); 2 Research Center Nutraceuticals and Food for Health, University of Pisa, Via del Borghetto 80, 56124 Pisa, Italy

**Keywords:** dairy sheep, gastrointestinal parasites, nitrogen excretion, phosphorus excretion, *Eimeria* spp., environmental sustainability

## Abstract

In sheep farming, gastrointestinal parasites can be responsible for significant reductions in animal health and production. Nitrogen (N) and phosphorus (P) fecal excretions are the main determining factors for N_2_O emissions from manure management and may pose other environmental problems, such as the acidification and eutrophication of natural habitats. By using the Mini-FLOTAC technique on fecal samples from sheep of different ages and physiological status from 19 dairy sheep farms in Tuscany (central Italy), gastrointestinal parasite infections were evaluated. The animal N and P fecal contents were also assessed, with the aim of evaluating possible relationships between the identified parasites and the environmental sustainability of the examined farms. The obtained results showed that *Eimeria* spp. (86.36%) and gastrointestinal strongyle (54.55%) infections are prevalent in the examined farms. Moreover, significantly higher (*p* ≤ 0.05) P and *Eimeria* oocyst/gram-of-feces (OPG) values were found in fecal samples from animals < 1 year of age, and a significant (*p* ≤ 0.05) positive correlation resulted between N content and *Eimeria* OPG in fecal samples from animals in the first month of lactation. The findings from this study suggest for the first time that *Eimeria* spp. infections may have an impact on the environmental sustainability of sheep farming.

## 1. Introduction

In Europe, the sheep dairy sector has a great relevance, providing about 40% of global sheep milk production, and it is concentrated largely in France, Greece, Spain, and Italy [1,2]. In the Mediterranean area, dairy sheep farms are mainly based on local breeds reared under extensive or semi-extensive management [3].

Gastrointestinal parasites, mainly gastrointestinal nematodes (GINs) and coccidia (*Eimeria* spp.), are included among the most widespread and important pathogens of the gastrointestinal tract in domestic ruminants worldwide [4,5]. In infected animals, these parasites can be responsible for severe clinical signs and significant reductions in production in clinical and subclinical infected animals, with consequent important economic losses [6,7]. Reduced productivity may arise from a combination of anorexia and reduced efficiency of resource use for production purposes [8].

GIN infections can lead to substantial changes in the digestive tract of small ruminants, such as increased cell turnover, changes in permeability, changes in pH, altered secretory activities, and inhibited gastric acid production [9]. Consequently, GIN infections may be responsible for an imbalance of nutrients and a negative impact on feed intake, growth and weight gain, fertility, and milk quality and quantity [6,10]. Moreover, the complex interactions between hosts and their gut microbiomes may be disrupted by nematode-induced changes in the intestinal tract [11].

Coccidia (*Eimeria* spp.) cause damage and necrosis in the intestinal epithelial cells, villous atrophy, and destruction or hyperplasia of the crypts [12,13]. Moreover, coccidian infections may strongly interact with the digestive microbiota, as the presence of the digestive microflora has been found essential to the development of parasite pathogenic expression [14]; coccidia may cause a massive change to the digestive microflora [7,15], and co-infections caused by coccidia and secondary bacterial infections are very frequent in sheep compared to *Eimeria* mono-infections [16,17].

The global livestock sector contributes a 14.5% to anthropogenic greenhouse gas (GHG) emissions, playing an important role in climate change, and small ruminants account for 6.5% of these CO_2_-equivalent emissions [18]. The major GHG sources from ruminants are methane (CH_4_) and nitrous oxide (N_2_O), which are primarily produced through enteric fermentation and manure management [19].

Nitrogen (N) excretion is considered the main determining factor for N_2_O emissions from manure management, an important GHG with a characterization factor 265 times greater for global warming potential than CO_2_ over a 100-year period [20], as reported in Table 1. N and phosphorus (P) excreted by animals also comprise the source of other types of emissions in air, soil, and water, such as ammonia (NH_3_), nitrogen oxides (NOx), nitrate (NO_3_), and phosphate (PO_4_^−^), which pose other environmental problems, such as the eutrophication (Table 1) of natural habitats [18,21]. 

High N excretions may also cause acidification (Table 1) of the environment because NOx and NH_3_ emissions lead to releases of hydrogen ions (H^+^), which contribute to the acidification of soils and water, resulting in forest decline and lake acidification [22]. 

**Table 1:** Main categories of potential environmental impacts of the emissions derived from nitrogen and phosphorous excreta in animal manure.

Emissions	Characterization Factor	Environmental Impact
CH_4_	28	Global warming potential, expressed as kg CO_2_ eq [23]
N_2_O	265	
NO_3_	0.1	Eutrophication, expressed as kg PO_4_^−^ eq [24]
P_2_O_3_	3.06	
NH_3_	1.6	Acidification, expressed as kg SO_2_ eq [25]
NOx	0.76	

Feed intake levels, feed composition, feed efficiency, and gut microflora are considered the primary factors affecting ruminant GHG emissions [26]. Reduced feed intake and feed efficiency caused by gastrointestinal dysfunction has been recognized as major contributors to increased GHG from livestock [27]. 

Therefore, gastrointestinal parasite infections may have the potential to impact livestock GHG emissions, as well as N and P excretions. Indeed, some recent studies have reported an increase in GHG emissions in sheep infected with GIN. Kenyon et al. [28] reported 10% higher emissions of CO_2_ eq per kg of weight gain in grazing lambs exposed to natural GIN infection under different anthelmintic treatments. Also, Fox et al. [29] recorded a 33% increase in methane yield (g CH_4_/kg dry matter intake) in lambs infected with larvae of *Teladorsagia circumcincta*, an abomasal GIN species. Finally, it was reported that the trickle infection of ewes with *T. circumcincta* increased the calculated GHG intensity for enteric (+11%) and manure (+32%) CH_4_ and N_2_O (+30%), for a total increase of 16% in the global warming potential [8].

The aim of this study was to evaluate possible relationships between gastrointestinal parasite infections and the environmental sustainability of dairy sheep farms in Tuscany (central Italy) by assessing the occurrence, frequency, and taxa of gastrointestinal parasites and N and P contents in fecal samples from animals on the examined farms.

## 2. Materials and Methods

### 2.1. Farms and Animals

A total of 19 dairy sheep farms were selected and analyzed. Farms differed in geographic area, flock size, breed reared (French Lacaune, Sardinian, Massese, and Comisana breeds), and rearing system. Specifically, farm locations and farming systems were chosen to be representative of the sheep milk production sector in the area examined. In fact, in Tuscany, most dairy sheep farms are in the southeastern area [30]. Therefore, 14 farms in the southeast, 3 in the northwest, and 2 in the center areas of Tuscany were selected. Moreover, the extensive farming system, in which sheep have access to pasture from 6 to 12 h per day, is prevalent in Tuscany, but there are also some intensive settings, where animals are stabled all day long. Therefore, 13 extensive and 6 intensive farms were selected. Flock sizes ranged from 101 to 300 heads (7 farms), 301 to 500 heads (6 farms), and above 500 heads (6 farms) (Table 2).

For each farm, data regarding liters of milk produced per head per year were registered.

### 2.2. Sampling and Parasitological Analysis

Individual fresh fecal samples were collected from each main animal category, i.e., animals for replacement (≥2 months and ≤1 year in age) and animals older than one year in age. Samples from these latter animals were further divided into samples taken from lactating ewes in the first month of lactation, lactating ewes after the first month of lactation, and dry ewes. On eight farms, it was not possible to collect samples from the replacement animals for two main reasons: only young animals less than 2 months old were present on the farm, or replacement animals were kept on a different farm site from that of adult animals, and the farm owners did not take us to these sites. A total of 220 individual samples were collected. Animals sampled in this study showed a body condition score (BCS) from 2.5 to 3 according to Russel et al. [31]. About 5 g of five individual fecal samples from the same animal category and farm were mixed to form pooled fecal samples, which were homogenized before analysis. The usefulness of pooled sheep fecal samples as a valid strategy procedure for the identification of gastrointestinal parasites in ruminants was previously reported [32,33]. A total of 44 pooled stool samples, including 33 pools from sheep older than one year in age and 11 pools from animals for replacement (Table 2), were examined via the Mini-FLOTAC technique using a low-density flotation solution (saturated NaCl solution, specific gravity = 1.200) to detect the presence and count the number of worm eggs and *Eimeria* spp. oocysts with a detection limit of 5 eggs/oocysts per gram (EPG/OPG) of feces [34]. A commercial rapid immunoassay (Rida Quick^®^ *Cryptosporidium*/*Giardia* Combi, R-Biopharm, Darmstadt, Germany) was also used for the detection of fecal antigens of *Giardia duodenalis* and *Cryptosporidium* spp. 

### 2.3. N and P Analysis 

All the pooled stool samples (n. 44) were analyzed in duplicate for N and P content using the methods suggested by the Association of Official Analytical Chemist [35]. Specifically, the Kjeldahl method was used for the evaluation of N content [35], while the evaluation of P content was carried out after digestion of samples with HNO_3_ and HClO_4_, and P was then quantified with a colorimetric method [35] using an ultraviolet–visible (UV–Vis) spectrophotometer (JASCO V-530, JASCO Europe S.r.l., Cremella, Como, Italy).

### 2.4. Statistical Analysis 

Differences in parasite frequency were statistically evaluated using a χ^2^ test, considering the three different flock sizes, the two rearing systems, and the two animal age categories (above or below one year of age).

Differences among gastrointestinal strongyle/coccidia quantitative data (EPG/OPG) were statistically analyzed with one-way ANOVA considering the variables of flock size, rearing system, age of animals, breed, and five different parameters: N and P content in g/100 g of feces, *Eimeria* spp. OPG, gastrointestinal strongyles EPG, and liters of milk produced per head per year.

Correlation analysis between N and P content and *Eimeria* spp. OPG and gastrointestinal strongyle EPG according to the different animal ages and physiological stages, i.e., lactating ewes in the first month of lactation, lactating ewes after the first month of lactation, dry ewes, and replacement sheep, and between *Eimeria* spp. OPG or gastrointestinal strongyle EPG and milk production, was also performed.

The statistical significance was set at *p* ≤ 0.05, and the analysis was carried out with JMP^®^ (Version 5.0) program.

## 3. Results

Overall, all 19 farms examined were found positive for more than one gastrointestinal parasitic species. As showed in Table 3, among the identified parasites, *Eimeria* spp. (86.36%, 38/44), and gastrointestinal strongyles (54.55%, 24/44), were found to be prevalent. Gastrointestinal strongyles were found to be significantly more prevalent in animals from extensive farms (*p* ≤ 0.0001) and older than one year in age (*p* ≤ 0.05).

The other parasites identified in this study were *Strongyloides papillosus* (22.72%, 10/44), *G. duodenalis* (9%, 4/44), *Moniezia* spp. (*Moniezia benedeni* and *Moniezia expansa*, 4.55%, 2/44), and *Trichuris* spp. (2.27%, 1/44) (Table 3).

Positivity for *G. duodenalis* was evidenced only on two farms, both characterized by a flock size over 500 heads and an intensive rearing system. The animals that tested positive were those in the first month of lactation and 2-month-old replacement in the first farm, and 2–4-month-old replacement in the second. Moreover, the occurrence of *G. duodenalis* was found to be significantly higher on intensive farms and in young animals (*p* ≤ 0.05). 

*Moniezia* spp. infection was found in lactating adult animals on two extensive farms of different flock sizes (101–300 and 301–500 heads). 

Only one pooled sample from dry sheep on a 101–300-head farm with an intensive rearing system tested positive for *Trichuris* spp.

No significant differences were found between breeds for any of the parameters considered.

Comparison between farms with different flock sizes showed a significantly higher (*p* ≤ 0.05) gastrointestinal strongyle EPG number in medium-sized farms between 301 and 500 heads (Table 4).

A significantly (*p* ≤ 0.001) higher milk production in terms of liters per head per year was found on farms with more than 500 heads. Finally, a significant higher (*p* ≤ 0.05) N content was found in fecal samples from animals on farms with between 101 and 300 heads. 

From the comparison of the different sheep farm rearing systems, animals with access to pasture had significantly higher (*p* ≤ 0.05) GIN EPG counts than animals raised in intensive farming systems.

Finally, fecal samples from animals less than one year old showed significantly higher (*p* ≤ 0.05) P and *Eimeria* OPG values than those from animals more than one year old.

**Figure 1:**
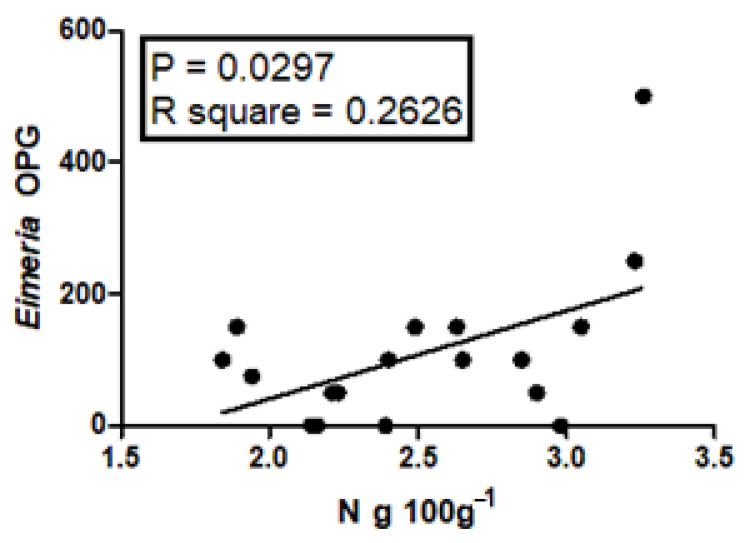
Correlation between N and *Eimeria* OPG in fecal samples of sheep in the first month of lactation.

Correlation analysis between N or P content and *Eimeria* spp. OPG or gastrointestinal strongyle EPG in fecal samples of animals at different physiological stages revealed a significant (*p* ≤ 0.05) positive correlation only between N and *Eimeria* OPG in fecal samples from animals in the first month of lactation (Figure 1). 

No significative results were obtained from the correlation between *Eimeria* spp. OPG or gastrointestinal strongyle EPG and milk production.

## 4. Discussion

Except for a recent study on the occurrence of *Cryptosporidium* spp. infections on sheep farms on the Italian island of Sardinia [36], recent data on sheep gastrointestinal parasites in Italy are lacking. The present study investigated the occurrence of gastrointestinal helminths and protozoa on dairy sheep farms in Tuscany, central Italy, and evaluated the possible impact of identified parasites on some environmental aspects of the sheep dairy sector in this area. 

In agreement with most of the studies on sheep gastrointestinal parasites in Europe [37,38], gastrointestinal strongyles and coccidia were found prevalent in sheep samples from examined dairy farms, confirming the importance of these gastrointestinal parasites in the dairy sheep sector. As previously reported [4,39], frequency and intensity of coccidian infections were found prevalent in young animals and on intensive farms, while gastrointestinal strongyles were found prevalent and at higher intensity in grazing sheep from extensive farms.

Among other protozoan and helminth infections identified at parasitological analysis, *S. papillosus* and *G. duodenalis* infections were found with a lesser frequency compared to gastrointestinal strongyles and coccidia. Again, these findings agree with those reported in recent studies performed in other European Mediterranean countries, such as in Greece, showing about 30% and 7.1% of sheep flocks positive for *G. duodenalis* and *S. papillosus*, respectively [37,38,40]. The cestodes *M. benedeni* and *M. expansa* and the nematode *Trichuris* spp. were seldom observed on the examined farms, especially this latter parasite, which was found on a single intensive farm. Contrary to what was recently observed in dairy sheep in Sardinia, in this study, none of the farms examined tested positive for *Cryptosporidium* spp. Nonetheless, it should be considered that *Cryptosporidium* spp. mainly infect lambs in the first month of life [36], while, in this study, animals older than one month of age were examined.

N and P contents in animal faeces were selected as indicators of the environmental impact of dairy sheep farming in this study. In fact, high levels of faecal N and P excretions may cause different types of emissions, into air, water, and soil [21,41]. 

Although N and P excretions constitute important factors for the level of ruminant farm emissions [41], the analysis of the N and P contents in animal faeces is an aspect that is still rarely considered [42]. Moreover, there are fewer reported data for sheep excretal outputs than for those of other livestock species, and even when nutritional studies are carried out, measurements of the mineral composition of dung and urine are generally not performed [42]. 

Interesting data were found in this study regarding the significant higher P contents and *Eimeria* OPG number found in fecal samples from animals less than one year old, and the significant positive correlation observed between N content and *Eimeria* OPG number in fecal samples from animals in the first month of lactation.

Compared to older sheep who acquire some degree of immunity to the infection, younger animals are considered the most at-risk age category for coccidiosis, in terms of higher prevalence and intensity of *Eimeria* spp. infections [5]. Lambs acquire the infection via the ingestion of mature *Eimeria* spp. oocysts contaminating food, water, and the farm environment [5]. Environmental contamination arises mainly from fecal *Eimeria* spp. oocysts shed by severely infected animals and by ewes in the periparturient period, but also by chronic and subclinical infected adult animals [5,43,44]. The results of this study show a significantly higher *Eimeria* OPG in younger animals compared to older animals, which is indicative of more severe infections in these animals, confirming previous data suggesting lambs as the main *Eimeria* spp. oocyst shedder on sheep farms due to a higher intensity of coccidian infections [7,45]. 

In severe infections, coccidia may be responsible for a significant reduction in intestinal function and massive alteration of the digestive microbiota due to severe intestinal pathological lesions [7,12,17]. Therefore, it is plausible that the higher P excretion observed in young animals shedding the higher numbers of *Eimeria* spp. OPG may be the result of intestinal disfunction and alterations in the intestinal microflora caused by more severe coccidian infections in this animal age category.

On the other hand, it is known that ewes may shed *Eimeria* spp. oocysts due to the peri-parturient relaxation of immunity [39], which, in dairy sheep, occurs mainly in late pregnancy and lasts for about three–four weeks after parturition [37,46]. Nevertheless, the peri-parturient excretion of *Eimeria* spp. oocysts implies that these protozoa are reproducing in the intestine of peri-parturient ewes with consequent possible intestinal pathological alterations, thus providing a possible explanation for the significant positive correlation observed in this study between N content and *Eimeria* OPG number in fecal samples from animals in the first month of lactation. Moreover, it is also known that the periparturient relaxation of immunity can also be associated with an increase in other parasites, especially gastrointestinal strongyles [8], but also *G. duodenalis* [47] and *S. papillosus* [48]. Indeed, ewes in the first month of lactation comprised an animal category found infected with *G. duodenalis* on one of the two farms scored *G. duodenalis*-positive in this study. Therefore, it is also possible that these other identified parasites may have concurred to worsen the intestinal lesions/disfunction caused by coccidia in sheep in the first month of lactation.

In agreement with results obtained in some previous studies on small ruminants [49,50], no significant data were obtained for the correlation between the *Eimeria* spp. OPG or gastrointestinal strongyle EPG number and the quantity of milk produced per head. However, negative effects on the milk composition were not evaluated in this study, while some studies report that GIN infections may have a negative impact on milk quality [6,10,49].

Conversely, significant differences were observed between farms of different sizes according to the milk yield. Specifically, farms with more than 500 heads had more successful milk production per animal, while lower milk production was observed in smaller farms with between 101 and 300 heads. 

On smaller farms, a lower milk production was also found to be associated with a higher N content in sheep feces. Consequently, a lower efficiency on smaller dairy sheep farms in Tuscany was seen in this study, as they produce less milk per head while dispersing higher N into the environment through animal fecal excreta. These findings agree with previous data reported in dairy cattle [19]. Nevertheless, eight out of nine smaller farms examined in this study were extensive farms, on which gastrointestinal strongyles were found to be significantly more prevalent compared to on intensive farms. As specified above, gastrointestinal strongyles may be able to reduce milk yield in sheep [6,10,50]. Although no significant correlations were found this study between gastrointestinal strongyle EPG counts and the quantity of milk produced per head, the possibility that gastrointestinal parasites may have contributed to the lower milk production and the higher N content in sheep from smaller farms cannot, therefore, be ruled out. 

## 5. Conclusions

The results of this study confirm the frequency and importance of gastrointestinal parasites, mainly gastrointestinal strongyles and coccidia, on sheep farms. Moreover, this study reports new data on the environmental sustainability of the dairy sheep sector.

To the best of our knowledge, this is the first study that evaluated gastrointestinal parasite infections in relation to nitrogen and phosphorus faecal content as indicators of the environmental sustainability of dairy sheep farming. Despite the limitation represented by the low number of samples/farms examined in this study, the obtained data are suggestive of a possible role of *Eimeria* spp. infections in the higher excretion of phosphorus in young animals, and of nitrogen in periparturient ewes.

Furthermore, this is the first study suggesting that coccidian (*Eimeria* spp.) infections may have an impact on the environmental sustainability of sheep farms, since the previous data only concerned gastrointestinal nematodes. Further studies are needed to confirm these results, and to evaluate whether the data we obtained can be linked to infections caused by more pathogenic *Eimeria* species or to higher GHG emissions in infected sheep. 

## Figures and Tables

**Table 2:** Flock sizes and breeding systems of the 19 dairy sheep farms analyzed in this study.

Farm n.	Flock Size	Rearing System	N. Pooled Fecal Samples *	
			<1 Year **	>1 Year ***
* 1	301–500 heads	I	1	2
2	>500 heads	I	1	2
3	>500 heads	I	1	1
4	>500 heads	I	1	2
5	101–300 heads	E	-	2
6	101–300 heads	E	1	2
7	301–500 heads	E	1	1
8	301–500 heads	E	-	3
9	301–500 heads	E	1	1
10	101–300 heads	I	1	2
11	301–500 heads	E	-	1
12	>500 heads	I	1	3
13	101–300 heads	E	-	2
14	101–300 heads	E	-	2
15	>500 heads	E	1	1
16	>500 heads	E	-	2
17	101–300 heads	E	1	1
18	301–500 heads	E	-	2
19	101–300 heads	E	-	1

I: intensive; E: extensive; *: pools composed of 5 individual fecal samples; **: samples from animals ≥ 2 months and ≤1 year in age; *** samples from animals > 1 year in age and including at least 1 pooled sample from lactating ewes in the first month of lactation/farm.

**Table 3:** Frequency of gastrointestinal parasites detected via parasitological analysis of pooled fecal samples from 19 dairy farms in Tuscany (central Italy).

Parasite	Overall	Flock Size			Rearing System		Age of Animals	
		101–300	301–500	>500	Intensive	Extensive	<1 Year	>1 Year
*Eimeria* spp. (Coccidia)	86.36% (38/44)	94.12% (16/17)	75.00% (9/12)	86.66% (13/15)	86.95% (20/23)	85.71% (18/21)	100% (11/11)	81.81% (27/33)
Gastrointestinal strongyles	54.55% (24/44)	41.17% (7/17)	75.00% (9/12)	53.33% (8/15)	26.09% (6/23)	85.71% (18/21)	27.27% (3/11)	63.63% (21/33)
*Strongyloides papillosus*	22.72% (10/44)	29.41% (5/17)	16.66% (2/12)	20.00% (3/15)	26.09% (6/23)	19.05% (4/21)	18.18% (2/11)	24.24% (8/33)
*Giardia duodenalis*	9.00% (4/44)	0.00% (0/17)	0.00% (0/12)	26.66% (4/15)	17.39% (4/23)	0.00% (0/21)	27.27% (3/11)	3.03% (1/33)
*Moniezia* spp.	4.55% (2/44)	5.88% (1/17)	8.33% (1/12)	0.00% (0/15)	0.00% (0/23)	9.52% (2/21)	0.00% (0/11)	6.06% (2/33)
*Trichuris* spp.	2.27% (1/44)	5.88% (1/17)	0.00% (0/12)	0.00% (0/15)	4.35% (1/23)	0.00% (0/21)	0.00% (0/11)	3.03% (1/33)

**Table 4:** Variation in N and P content (g/100 g of feces), *Eimeria* OPG, gastrointestinal strongyle (GIS) EPG, and production of milk (liter head^−1^ year^−1^) according to the flock size, rearing system, and age of sheep.

Parameter	Flock Size			Rearing System		Age of Animals	
	101–300	301–500	>500	Intensive	Extensive	<1 Year	>1 Year
N	2.59 ± 0.383 ^a^	2.41 ± 0.415 ^ab^	2.14 ± 0.485 ^b^	2.33 ± 0.470	2.46 ± 0.454	2.33 ± 0.421	2.40 ± 0.472
P	0.58 ± 0.172	0.57 ± 0.108	0.45 ± 0.125	0.53 ± 0.162	0.49 ± 0.141	0.63 ± 0.199 ^a^	0.48 ± 0.125 ^b^
*Eimeria* OPG	2283.33 ± 6957.446	413.89 ± 843.937	2837.50 ± 8492.033	3454.69 ± 8447.538	281.67 ± 716.938	7689.29 ± 12,315.013 ^a^	236.46 ± 561.973 ^b^
GIS EPG	43.75 ± 85.480 ^b^	511.11 ± 604.060 ^a^	35.00 ± 78.351 ^b^	3.13 ± 28.115 ^b^	361.67 ± 520.925 ^a^	0.00 ± 0.000	228.125 ± 433.221
Milk	130.33 ± 64.791 ^B^	195.78 ± 74.095 ^B^	345.90 ± 143.580 ^A^	242.63 ± 191.977	193.53 ± 63.710	-	-

Values are presented as mean ± standard deviation; ^a,b^ means with a different superscript letter within a row differ significantly *p* ≤ 0.05; ^A,B^ means with a different superscript letter within a row differ significantly *p* ≤ 0.001.

## Data Availability

Raw data supporting the conclusions of this study are available from the authors upon request.

## References

[1] de Rancourt M., Fois N., Lavín M.P., Tchakérian E., Vallerand F. (2006). Mediterranean sheep and goat production: An uncertain future. Small Rumin. Res..

[2] Odintsov Vaintrub M., Levit H., Chincarini M., Fusaro I., Giammarco M., Vignola G. (2021). Review: Precision livestock farming, automats and new technologies: Possible applications in extensive dairy sheep farming. Animal.

[3] Altomonte I., Conte G., Serra A., Mele M., Cannizzo L., Salari F., Martini M. (2019). Nutritional characteristics and volatile components of sheep milk products during two grazing seasons. Small Rumin. Res..

[4] Maurizio A., Perrucci S., Tamponi C., Scala A., Cassini R., Rinaldi L., Bosco A. (2023). Control of gastrointestinal helminths in small ruminants to prevent anthelmintic resistance: The Italian experience. Parasitology.

[5] Bangoura B., Bardsley K.D. (2020). Ruminant Coccidiosis. Vet. Clin. N. Am. Food Anim. Pract..

[6] Charlier J., Bartley D.J., Sotiraki S., Martinez-Valladares M., Claerebout E., von Samson-Himmelstjerna G., Thamsborg S.M., Hoste H., Morgan E.R., Rinaldi L. (2022). Anthelmintic resistance in ruminants: Challenges and solutions. Adv. Parasitol..

[7] Chartier C., Paraud C. (2012). Coccidiosis due to *Eimeria* in sheep and goats, a review. Small Rumin. Res..

[8] Houdijk J.G.M., Tolkamp B.J., Rooke J.A., Hutchings M.R. (2017). Animal health and greenhouse gas intensity: The paradox of periparturient parasitism. Int. J. Parasitol..

[9] Louie K., Vlassoff A., Mackay A.D. (2007). Gastrointestinal nematode parasites of sheep: A dynamic model for their effect on liveweight gain. Int. J. Parasitol..

[10] Hoste H., Meza-Ocampos G., Marchand S., Sotiraki S., Sarasti K., Blomstrand B.M., Williams A.R., Thamsborg S.M., Athanasiadou S., Enemark H.L. (2022). Use of agro-industrial by-products containing tannins for the integrated control of gastrointestinal nematodes in ruminants. Parasite.

[11] Zaiss M.M., Harris N.L. (2016). Interactions between the intestinal microbiome and helminth parasites. Parasite Immunol..

[12] Gregory M.W., Catchpole J. (1987). Ovine coccidiosis: Pathology of *Eimeria ovinoidalis* infection. Int. J. Parasitol..

[13] Taylor M.A., Catchpole J., Marshall J., Marshall R.N., Hoeben D. (2003). Histopathological observations on the activity of diclazuril (Vecoxan®) against the endogenous stages of *Eimeria crandallis* in sheep. Vet. Parasitol..

[14] Gouet P., Yvore P., Naciri M., Contrepois M. (1984). Influence of digestive microflora on parasite development and the pathogenic effect of *Eimeria ovinoidalis* in the axenic, gnotoxenic and conventional lamb. Res. Vet. Sci..

[15] Lu C., Yan Y., Jian F., Ning C. (2021). Coccidia-Microbiota Interactions and Their Effects on the Host. Front. Cell. Infect. Microbiol..

[16] Al-Neama R.T., Bown K.J., Blake D.P., Birtles R.J. (2021). Determinants of *Eimeria* and *Campylobacter* infection dynamics in UK domestic sheep: The role of co-infection. Parasitology.

[17] Bangoura B., Bhuiya M.A.I., Kilpatrick M. (2022). *Eimeria* infections in domestic and wild ruminants with reference to control options in domestic ruminants. Parasitol. Res..

[18] Gerber P.J., Steinfeld H., Henderson B., Mottet A., Opio C., Dijkman J., Falcucci A., Tempio G. (2013). Tackling Climate Change through Livestock—A Global Assessment of Emissions and Mitigation Opportunities.

[19] Singaravadivelan A., Sachin P.B., Harikumar S., Vijayakumar P., Vindhya M.V., Farhana F.M.B., Rameesa K.K., Mathew J. (2023). Life cycle assessment of greenhouse gas emission from the dairy production system—Review. Trop Anim Health Prod..

[20] IPCC—International Panel on Climate Change (2007). Climate Change 2007: Synthesis Report.

[21] Nemecek T., Kägi T. (2007). Life Cycle Inventories of Agricultural Production Systems.

[22] FAO—Food and Agriculture Organization of the United Nations (2016). Greenhouse Gas Emissions and Fossil Energy Use from Small Ruminant Supply Chains: Guidelines for Assessment.

[23] IPCC—International Panel on Climate Change (2019). Chapter 11: N_2_O emissions from managed soils, and CO_2_ emissions from lime and urea application. Agriculture, Forestry and Other Land Use.

[24] Heijungs R., Guinee J.B., Huppes G., Lankreijer R.M., Udo de Haes H.A., Wegener-Sleeswijk A., Ansems A.M.M., Eggels P.G., van Duin R., de Goede H.P. (1992). Environmental Life Cycle Assessment of Products: Guide and Backgrounds.

[25] Huijbregts M. (1999). Life-cycle Impact Assessment of Acidifying and Eutrophying Air Pollutants. Calculation of Equivalency Factors with RAINS-LCA.

[26] Lascano E.A., Cárdenas E.A. (2010). Alternatives for methane emission mitigation in livestock systems. Rev. Bras. De Zootec..

[27] Basarab J.A., Beauchemin K.A., Baron V.S., Ominski K.H., Guan L.L., Miller S.P., Crowley J.J. (2013). Reducing GHG emissions through genetic improvement for feed efficiency: Effects on economically important traits and enteric methane production. Animal.

[28] Kenyon F., Dick J.M., Smith R.I., Coulter D.G., McBean D., Skuce P.J. (2013). Reduction in Greenhouse Gas Emissions Associated with Worm Control in Lambs. Agriculture.

[29] Fox N.J., Smith L.A., Houdijk J.G.M., Athanasiadou S., Hutchings M.R. (2018). Ubiquitous parasites drive a 33% increase in methane yield from livestock. Int. J. Parasitol..

[30] (2021). Italian National Livestock Registry. https://www.vetinfo.it/j6_statistiche/index.html#/report-pbi/29.

[31] Russel A.J.F., Doney J.M., Gunn R.G. (1969). Subjective assessment of body fat in live sheep. J. Agric. Sci..

[32] Rinaldi L., Levecke B., Bosco A., Ianniello D., Pepe P., Charlier J., Cringoli G., Vercruysse J. (2014). Comparison of individual and pooled faecal samples in sheep for the assessment of gastrointestinal strongyle infection intensity and anthelmintic drug efficacy using McMaster and Mini-FLOTAC. Vet. Parasitol..

[33] Maurizio A., Marchiori E., Tessarin C., Cassini R. (2023). Comparing pooled and individual samples for estimation of gastrointestinal strongyles burden and treatment efficacy in small ruminants. Vet. Parasitol..

[34] Bosco A., Kießler J., Amadesi A., Varady M., Hinney B., Ianniello D., Maurelli M.P., Cringoli G., Rinaldi L. (2020). The threat of reduced efficacy of anthelmintics against gastrointestinal nematodes in sheep from an area considered anthelmintic resistance-free. Parasit. Vectors.

[35] AOAC—Association of Official Analytical Chemist (2000). Official Methods of Analysis.

[36] Dessì G., Tamponi C., Varcasia A., Sanna G., Pipia A.P., Carta S., Salis F., Díaz P., Scala A. (2020). *Cryptosporidium* infections in sheep farms from Italy. Parasitol. Res..

[37] Lianou D.T., Arsenopoulos K.V., Michael C.K., Papadopoulos E., Fthenakis G.C. (2022). Protozoan Parasites in Adult Dairy Small Ruminants and Potential Predictors for Their Presence in Faecal Samples. Microorganisms.

[38] Lianou D.T., Arsenopoulos K.V., Michael C.K., Mavrogianni V.S., Papadopoulos E., Fthenakis G.C. (2023). Helminth Infections in Dairy Sheep Found in an Extensive Countrywide Study in Greece and Potential Predictors for Their Presence in Faecal Samples. Microorganisms.

[39] Platzer B., Prosl H., Cieslicki M., Joachim A. (2005). Epidemiology of *Eimeria* infections in an Austrian milking sheep flock and control with diclazuril. Vet. Parasitol..

[40] Tzanidakis N., Sotiraki S., Claerebout E., Ehsan A., Voutzourakis N., Kostopoulou D., Stijn C., Vercruysse J., Geurden T. (2014). Occurrence and molecular characterization of *Giardia duodenalis* and *Cryptosporidium* spp. in sheep and goats reared under dairy husbandry systems in Greece. Parasite.

[41] IPCC—International Panel on Climate Change (2019). Refinement to the 2006 IPCC Guidelines for National Greenhouse Gas Inventories, Chapter 10, Emissions From Livestock And Manure Management. Agriculture, Forestry and Other Land Use.

[42] Smith K.A., Frost J.P. (2000). Nitrogen excretion by farm livestock with respect to land spreading requirements and controlling nitrogen losses to ground and surface waters. Part 1: Cattle and sheep, Bioresour. Technol..

[43] Foreyt W.J. (1990). Coccidiosis and cryptosporidiosis in sheep and goats. Vet. Clin. N. Am. Food Anim. Pract..

[44] Carrau T., Silva L.M.R., Pérez D., Failing K., Martínez-Carrasco C., Macías J., Taubert A., Hermosilla C., de Ybáñez R.R. (2018). Associated risk factors influencing ovine *Eimeria* infections in southern Spain. Vet. Parasitol..

[45] Keeton S.T.N., Navarre C.B. (2018). Coccidiosis in Large and Small Ruminants. Vet. Clin. N. Am. Food Anim. Pract..

[46] Barger I.A. (1993). Influence of sex and reproductive status on susceptibility of ruminants to nematode parasitism. Int. J. Parasitol..

[47] Xiao L.H., Herd R.P., McClure K.E. (1994). Periparturient rise in the excretion of *Giardia* sp. cysts and *Cryptosporidium parvum* oocysts as a source of infection for lambs. J. Parasitol..

[48] Agyei A.D., Sapong D., Probert A.J. (1991). Periparturient rise in faecal nematode egg counts in west African dwarf sheep in southern Ghana in the absence of arrested strongyle larvae. Vet. Parasitol..

[49] Mavrot F., Hertzberg H., Torgerson P. (2015). Effect of gastro-intestinal nematode infection on sheep performance: A systematic review and meta-analysis. Parasit. Vectors.

[50] Cringoli G., Rinaldi L., Veneziano V., Mezzino L., Vercruysse J., Jackson F. (2009). Evaluation of targeted selective treatments in sheep in Italy: Effects on faecal worm egg count and milk production in four case studies. Vet. Parasitol..

